# Fast-Acting Chalcogen-Phosphoranes Inhibit Sensitive and Resistant *Plasmodium falciparum* Strains

**DOI:** 10.1021/acsomega.5c08794

**Published:** 2026-01-05

**Authors:** Igor M. R. Moura, Camila S. Barbosa, Giovana Rossi Mendes, Anna Caroline Campos Aguiar, Fabio C. Cruz, Paulo Henrique Menezes, Rafael Victorio Carvalho Guido

**Affiliations:** † São Carlos of Physics Institute, 117186 University of São Paulo (USP), 13566-590 São Carlos, SP, Brazil; ‡ Department of Microbiology, Immunology and Parasitology, 28105 Federal University of São Paulo (UNIFESP), Escola Paulista de Medicina, 04023-062 São Paulo, SP, Brazil; § Department of Pharmacology, 28105 Federal University of São Paulo (UNIFESP), Escola Paulista de Medicina, 04023-062 São Paulo, SP, Brazil; ∥ Department of Fundamental Chemistry, 28116 Federal University of Pernambuco, 50740-560 Recife, PE, Brazil

## Abstract

Malaria remains a major global health challenge with growing resistance to current antimalarial drugs. In this study, we report the antiplasmodial activity of eight sulfur-containing chalcogen-phosphorane analogues that show submicromolar potency against *Plasmodium falciparum* 3D7 strain (IC_50_s = 0.7–1.9 μM) and reasonable selectivity indexes (SI) over HepG2 and HEK293 cells (SI ≥ 12). Electron-withdrawing substituents on the aromatic ring improved potency by two- to 3-fold. Compound **5**, a representative compound of the series, showed fast-acting inhibitory activity that rapidly induced pyknotic nuclei, demonstrated no cross-resistance with standard antimalarials, and exhibited an additive interaction with artesunate. Stage-specific assays revealed pronounced activity against ring and trophozoite stages but reduced efficacy against schizonts, with longer exposure needed for maximal effects. In a murine *Plasmodium berghei* model, compound **5** showed modest in vivo activity but slightly improved the survival rate. These findings suggest that sulfur-containing chalcogen-phosphoranes represent an attractive series for hit-to-lead development.

## Graphical abstract

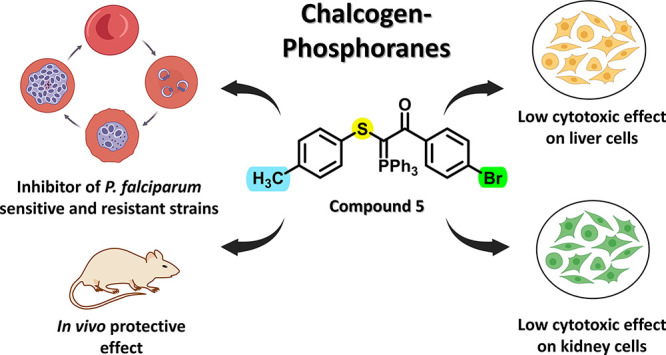

## Introduction

Malaria is a parasitic infectious disease caused by various species of *Plasmodium*, including *P. falciparum*, *P. vivax*, *P. ovale curtisi*, *P. ovale wallikeri*, *P. malariae*, and *P. knowlesi*. It is prevalent across tropical and subtropical regions and poses a significant public health issue due to high mortality rates. According to the World Health Organization (WHO), there were 263 million malaria cases and an estimated 597,000 deaths in 2023.^1^ Nearly 90% of malaria-related deaths occurred in Africa, with children under five and pregnant women being the most vulnerable groups. With the onset of the COVID-19 pandemic, decades of progress in malaria treatment were reversed and led to a significant escalation in malaria deaths in Sub-Saharan Africa in 2020.^1^ Therefore, malaria remains a primary health concern globally, underscoring the urgent need for effective control.

Over the past two decades, malaria control has relied mainly on insecticide-treated bed nets, rapid diagnosis, and artemisinin based combination therapies (ACTs).^1^ However, the emergence of parasites partially resistant to ACTs, now also present in Africa, poses a serious threat to malaria containment, necessitating the development of effective alternatives.^2–5^ This situation has spurred the search for new approaches to antimalarial drug discovery and development, leading to a significant expansion of the antimalarial lead portfolio in the 21st century.^6–10^

Chalcogen-phosphoranes are a class of phosphoranes containing chalcogen atoms (e.g., sulfur, selenium, or tellurium) bound to an sp^2^ carbon close to the phosphorus atom.^11^

Phosphorus-containing molecules play a crucial role in various life-sustaining pathways. In pharmaceuticals, these molecules are often designed to enhance drug-like properties. Among the different classes of phosphorus compounds, phosphonates are the most commonly found in drug-like molecules, such as fosmidomycin, an antibiotic known to inhibit the MEP pathway in *Plasmodium* parasites.^12–15^ Among phosphorus-containing molecules, phosphoranes represent a class of phosphorus compounds featuring five covalently bonded ligands, with the phosphorus atom existing in the oxidation state P­(V).^16,12^ Renowned for their utility in organic synthesis, phosphoranes play a crucial role in the Wittig reaction, serving as carbon-group donors endowed with diverse chemical properties.^11,17,18^ Despite their prominence in synthetic approaches, the investigation of the biological properties of phosphoranes is relatively limited. Few studies have reported the biological activity of phosphoranes, including the antimicrobial potential of phosphoranes, particularly those harboring five- and six-membered rings incorporating P­(V) within their structure.^19,20^

In this study, we report the evaluation of the antiplasmodial and cytotoxic activities of selected chalcogen-phosphoranes. Moreover, the results of a comprehensive parasitological study including in vitro and in vivo assays are described.

## Results and Discussion

### Thio-phosphoranes Show Antiplasmodial Activity and Reasonable Selectivity

The synthetic scheme of compounds **1**–**8** is indicated in Figure 1. The synthesis and structural characterization of compounds **1**–**8** are reported elsewhere.^11^

**Figure 1:**
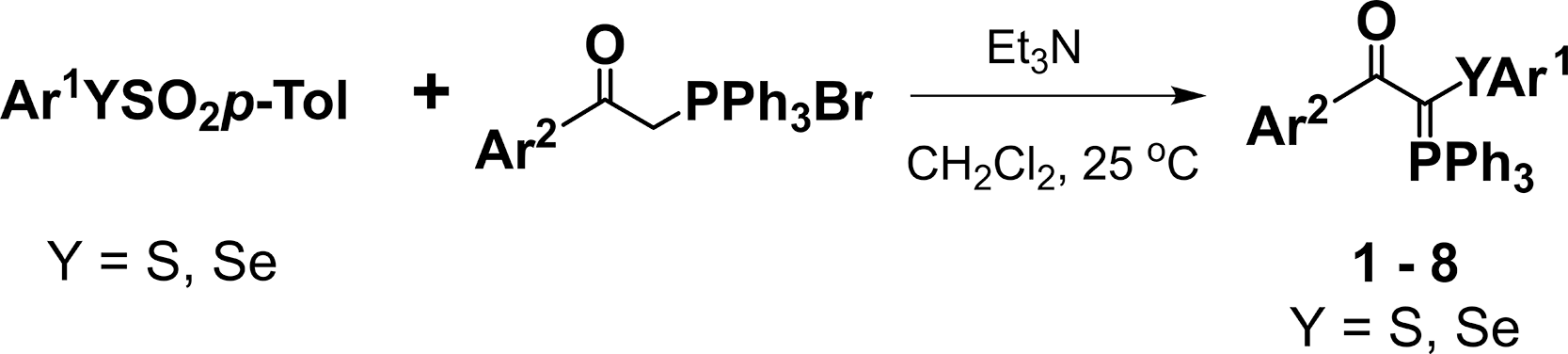
Synthetic scheme for the obtention of compounds **1**–**8**.

Eight chalcogen-phosphorane derivatives were subjected to antiplasmodial activity evaluation against the chloroquine-sensitive *P. falciparum* 3D7 strain (Table 1 and Figure S1). Among them, four analogues (**1**, **2**, **4**, and **8**) displayed moderate inhibitory activity (1 μM ≤ IC_50_^3D7^ ≤ 10 μM), while two (**3** and **5**) exhibited potent antiplasmodial activity (IC_50_^3D7^ < 1 μM). Notably, the presence of a sulfur atom in the phosphorane structure was favorable for inhibitory activity, resulting in analogues with moderate to potent antiplasmodial activities (**1**–**5**), whereas its substitution with a selenium atom resulted in a loss of inhibitory activity (**6**–**8**). For instance, compound **1**, a thio-phosphorane analog, showed moderate activity (IC_50_^3D7^ = 1.9 μM), while compound **6**, a seleno-phosphorane analog, exhibited poor antiplasmodial activity (IC_50_^3D7^ > 10 μM). The introduction of an electron-donating group (EDG), such as a *p*-methoxy substituent (**2**, IC_50_^3D7^ = 1.2 μM), was tolerated. Conversely, substitution with an electron-withdrawing group (EWG), such as a *p*-nitro substituent (**3**, IC_50_^3D7^ = 0.78 μM), resulted in a 2.5-fold increase in potency compared to **1**. Replacement with another EWG with reduced steric volume, such as a fluorine atom (**4**, IC_50_^3D7^ = 1.8 μM), did not enhance inhibitory activity. However, replacement with another halogen substituent with increased steric volume, such as bromine (**5**, IC_50_^3D7^ = 0.7 μM), led to a 3-fold increase in potency compared to **1**. These findings suggest that bulky EWG substituents at the *para* position of the phenyl group are favorable for the inhibitory activity of this series.

**Table 1:**
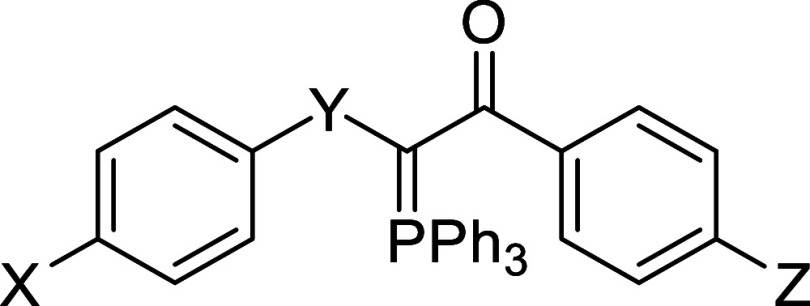
In Vitro Antiplasmodial Activity against Chloroquine-Sensitive *P. falciparum* (3D7 strain) and Cytotoxic Activity against HepG2 and HEK293 Cells of Chalcogen-Phosphoranes^a^

				inhibition @ 10 μM	IC_50_^3D7^ (μM)	CC_50_^HepG2^ (μM)		CC_50_^HEK293^ (μM)	
Cpd	X	Y	Z	mean ± SD	mean ± SD	mean ± SD	SI	mean ± SD	SI
**1**	Me	S	H	96 ± 2	1.9 ± 0.2	>50	>26	>50	>26
**2**	Me	S	OMe	97 ± 3	1.2 ± 0.1	24 ± 1	20	>50	>42
**3**	Me	S	NO_2_	93 ± 2	0.78 ± 0.05	38 ± 2	49	>50	>64
**4**	Me	S	F	94 ± 4	1.8 ± 0.5	22 ± 2	12	>50	>28
**5**	Me	S	Br	95 ± 4	0.7 ± 0.2	>25	>37	>50	>71
**6**	H	Se	H	48 ± 9	>10	nd		nd	
**7**	H	Se	OMe	40 ± 6	>10	nd		nd	
**8**	H	Se	NO_2_	85 ± 5	4.2 ± 0.5	24 ± 2	6	26 ± 13	6
**ART**					0.012 ± 0.003	nd		nd	

a ART = artesunate (positive control for inhibition); SI = selectivity index (CC_50_/IC_50_); nd = not determined.

The SAR investigation suggested that a sulfur atom in the structure of the chalcogen-phosphorane series is necessary for antiplasmodial activity. The substitution with a selenium atom in analogues **6**–**8** increases chemical reactivity due to the larger atomic size of selenium (115 pm) compared to sulfur (100 pm). This size difference results in weaker π-overlap in selenium-containing molecules, leading to higher reactivity than their sulfur-containing counterparts.^21,22^ However, increased reactivity was not observed under antiplasmodial assay conditions. Thus, the antiplasmodial activity measured for the chalcogen-phosphorane derivatives is not attributable to cytotoxic effects.

To further investigate the biological properties of these derivatives, we assessed the cytotoxic effects of the inhibitors with IC_50_^3D7^ values <10 μM against two cell lines, hepatocarcinoma (HepG2) and human embryonic kidney (HEK293) cells (Table 1 and Figures S2 and S3). The tested compounds exhibited comparable cytotoxic profiles in both HepG2 (CC_50_^HepG2^ ranging from 24 to >50 μM) and HEK293 (CC_50_^HEK293^ ranging from 26 to >50 μM) cells. HepG2 cells originate from human liver cancer, while HEK293 cells are derived from human embryonic kidney tissue. Both cell lines are commonly used in drug development to assess potential hepatic and renal toxicity.^23,24^ Notably, molecular substitutions aimed at enhancing antiplasmodial activity did not significantly affect cytotoxicity, indicating pronounced selectivity of these modifications toward antiplasmodial action (selectivity index, SI > 26). In drug discovery campaigns, selectivity index (SI) values greater than 10 are prioritized.^25^ In addition to their low cytotoxic effect on HepG2 and HEK293 cells (CC_50_ *s* > 22 μM), chalcogen-phosphoranes showed no hemolytic activity against fresh red blood cells (Figure S4).

### Thio-phosphoranes Showed No Cross-resistance with Standard Antimalarials

To evaluate the inhibitory potential of the chalcogen-phosphorane series against resistant *P. falciparum* strains, we selected compounds **1**, **3**, and **5** as representatives for the chemical series. These compounds were the most potent inhibitors and had low cytotoxic effects on liver and kidney cells. These compounds were assessed against a small representative panel of drug-resistant *P. falciparum* strains composed of Dd2 (resistant to chloroquine, sulfadoxine, pyrimethamine, mefloquine, and cycloguanil), TM90C6B (resistant to chloroquine, pyrimethamine and atovaquone), and 3D7^R^_MMV848 (a strain derived from 3D7 resistant to MMV692848, a PfPI4K inhibitor) strains.

To establish the resistance profile of the strains, pyrimethamine (PYR), atovaquone (ATV), and MMV692848 were employed as positive controls against Dd2, TM90C6B, and 3D7^R^_MMV848 strains, respectively. These antimalarial drugs were concurrently tested in the chloroquine-sensitive 3D7 strain to compare the resistance profiles and determine the resistance index (RI) values. In this context, a compound is deemed to exhibit a cross-resistance profile with the respective antimalarial drug if the IC_50_ value in the resistant strain is at least 3-fold greater than the IC_50_ value in the sensitive strain (RI > 3).^26^

PYR, ATV, and MMV692848 showed RI values greater than 185, 745, and 18 against Dd2, TM90C6B, and 3D7^R^_MMV848, respectively. Furthermore, artesunate (ART) exhibited comparable inhibitory activity against all strains evaluated (Table 2; Figures 2A and S5). These results confirmed the resistant profile of the representative *P. falciparum* strains. Compounds **1**, **3**, and **5** demonstrated comparable inhibitory potency values against the resistant strains, with RI varying from 1.0 to 2.1. These findings indicate the absence of cross-resistance between the chalcogen-phosphorane derivatives and the standard antimalarials.

**Table 2:** Inhibitory Activity and Resistance Index (RI) Values of 1, 3, and 5 and Standard Antimalarial Drugs against Sensitive (3D7) and Resistant (Dd2, TM90C6B, and 3D7^R^_MMV848) *P. falciparum* Strains (RI = IC_50_^strain^/IC_50_^3D7^)­^a^

	IC_50_ (μM)						
compound	3D7	Dd2	RI	TM90C6B	RI	3D7^R^_MMV848	RI
**1**	2.2 ± 0.3	2.1 ± 0.5	1.0 ± 0.2	2.3 ± 0.4	1.0 ± 0.2	2.3 ± 0.7	1.1 ± 0.3
**3**	1.4 ± 0.2	1.5 ± 0.5	1.1 ± 0.3	1.8 ± 0.2	1.3 ± 0.1	1.5 ± 0.4	1.1 ± 0.3
**5**	0.6 ± 0.1	0.9 ± 0.2	1.4 ± 0.4	1.3 ± 0.2	2.1 ± 0.3	0.7 ± 0.2	1.2 ± 0.4
**artesunate**	0.012 ± 0.003	0.007 ± 0.002	0.6 ± 0.2	0.008 ± 0.003	0.7 ± 0.2	0.012 ± 0.006	1.0 ± 0.5
**pyrimethamine**	0.0541 ± 0.0003	>10	>185	>10	>185	0.050 ± 0.001	0.93 ± 0.03
**atovaquone**	0.0013 ± 0.0004	0.0007 ± 0.0003	0.5 ± 0.2	>1	>745	0.0013 ± 0.0005	1.0 ± 0.3
**MMV692848**	0.17 ± 0.02	0.13 ± 0.03	0.8 ± 0.2	0.21 ± 0.06	1.3 ± 0.3	3.0 ± 0.2	18 ± 1

a (*N*,*n* = 3,2; mean ± SD). Values are mean ± standard deviation.

**Figure 2:**
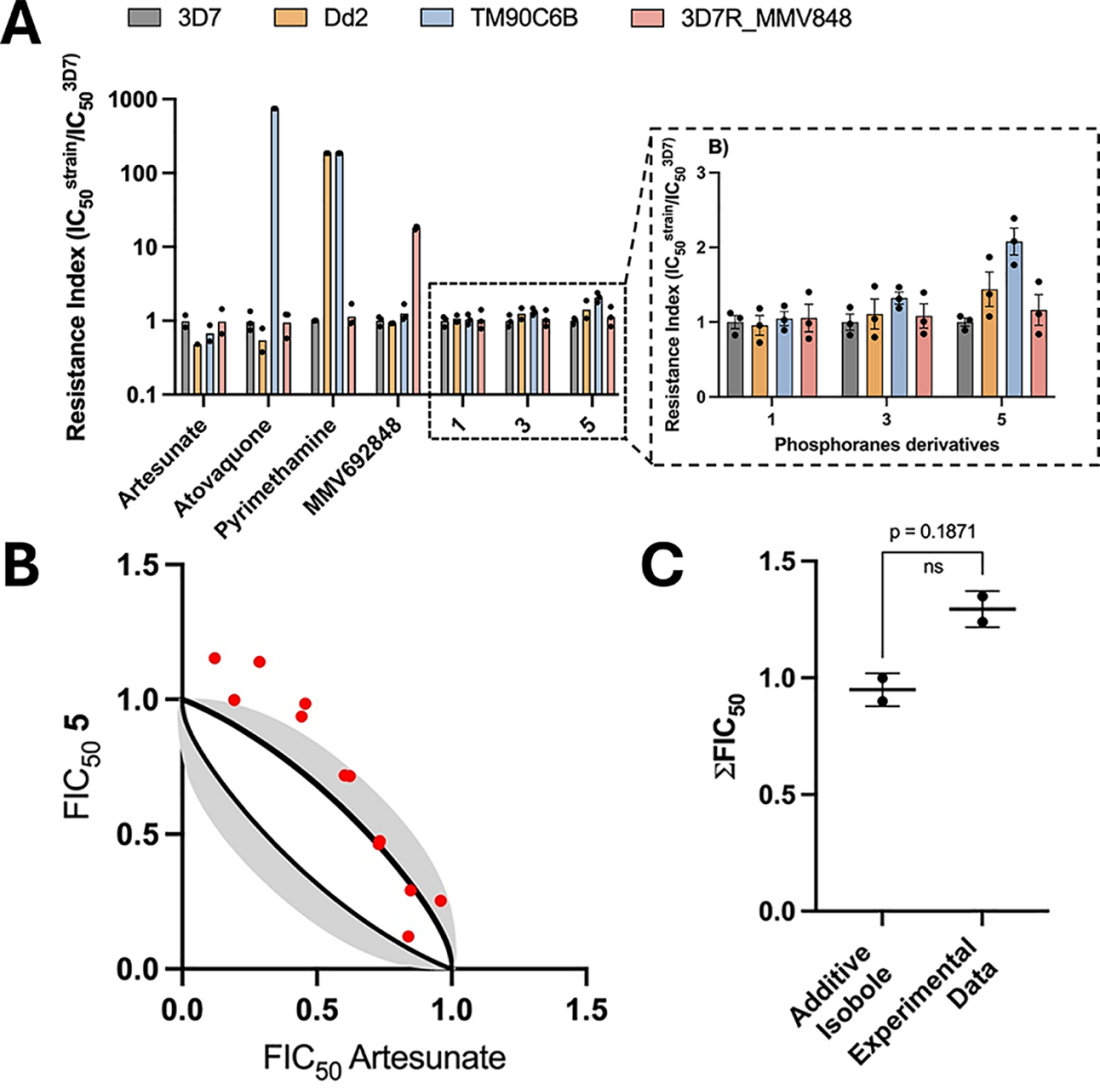
(A) Analysis of RI values of 1, 3, and 5 against a representative panel of resistant *P. falciparum* strains relative to the sensitive strain (*N*, *n* = 2–3, 2; mean ± SD). Insert: Amplification of the resistance index region (linear scale) for compounds **1**, **3**, and **5**. (B) Isobologram and additive isoboles calculated for the association between 5 and ART. The black lines represent the calculated additive isobole, with its respective standard deviation shown in gray, and experimental points are depicted as red dots. (C) Comparison between the ∑FIC_50_ values from the additive isobole and the experimental data. Values are mean ± standard deviation. Statistical significance was determined using a paired Student’s *t* test.

### Thio-phosphoranes Show an Additive Combination Profile with Artesunate

A combination assay was performed to assess the efficacy of **5** as a potential antiplasmodial partner with ART, an artemisinin derivative commonly used as a standard antimalarial agent, especially in artemisinin combination therapies. The nature of the drug interaction was characterized using fractional inhibitory concentration of IC_50_ (FIC_50_) values. In this analysis, FIC_50_ values, represented by red dots within the gray region of the isobole, indicate an additive profile; values above the gray area reflect antagoni**s**tic interactions, while those below suggest synergy (Figure 2B). Considering the experimental variability associated with the additive isobole and the data obtained, the combination of **5** and artesunate was classified as exhibiting additive behavior (Figure 2B). To quantify this interaction, the FIC index was calculated. A value of 1 denotes additivity, a value less than 1 indicates synergy, and a value greater than 1 represents antagonism. The computed ∑FIC index for **5** was 1.29 ± 0.08 with no statistical difference from the ∑FIC index for the additive isobole, suggesting an additive effect when combined with ART.

### Thio-phosphoranes Are Fast-Acting Inhibitors

To assess the speed-of-action profile of **5**, qualitative and quantitative analyses were conducted. The quantitative approach involved assessing the IC_50_ value of **5** at three different time points (24, 48, and 72 h) using ring-synchronized parasites. In these assays, we used ART and PYR as controls for fast- and slow-acting inhibitors, respectively (Figure 3). Fast-acting inhibitors typically exhibit consistent IC_50_ values across all exposure times, whereas slow-acting inhibitors display varying IC_50_ values, usually showing higher values at 24 h compared to subsequent time frames.^26,27^ Our findings revealed that the inhibitory activity of **5** showed no differences among 24, 48, and 72 h of exposure to the inhibitor, comparable to ART, thereby indicating that the compound acts as a fast-acting inhibitor (Figure 3A).

**Figure 3:**
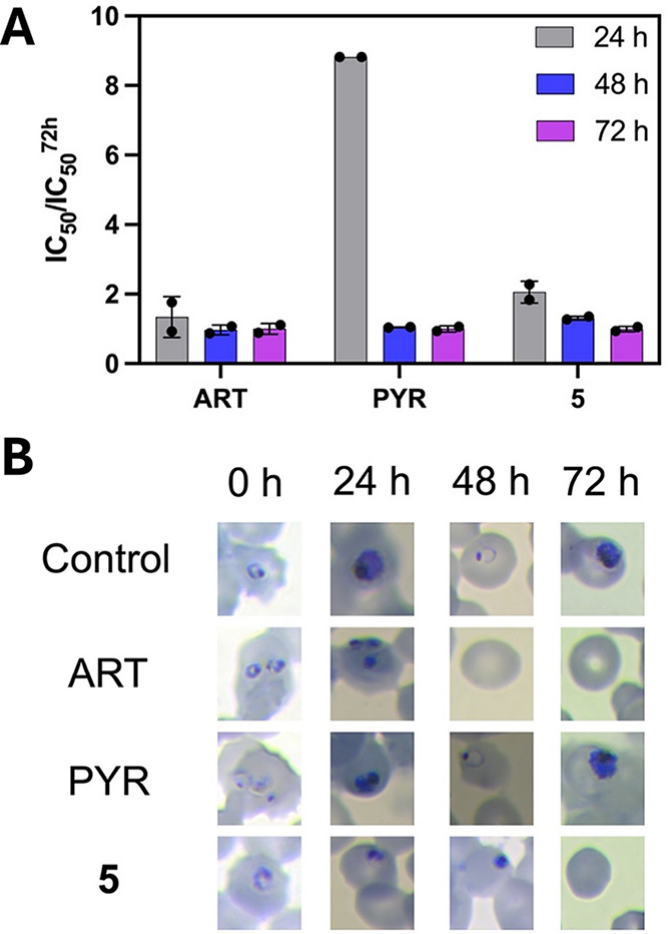
Speed-of-action assessment of 5. (A) IC_50_ ratios were determined at 24, 48, and 72 h. Artesunate (ART) and pyrimethamine (PYR) were used as fast- and slow-acting inhibitor controls, respectively. (B) Evaluation of the parasite morphological development over time in *P. falciparum* cultures stained with Giemsa. Data shows mean ± standard deviation (*N*, *n* = 2,2).

In the qualitative assay, we examined and compared the morphology of *P. falciparum* parasites over a 72-h period, assessing samples at 24, 48, and 72 h following 24 h of inhibitor exposure. Fast-acting inhibitors are effective at eliminating parasites within 24 h, while slow-acting compounds require extended exposure times to achieve comparable results. Given the parasite’s life cycle of 42–44 h, a consistent morphological development was observed (Figure 3B, control). ART, being a fast-acting antimalarial, effectively hindered parasite development within the initial 24 h of exposure, as evidenced by the presence of pyknotic nuclei (Figure 3B, ART). Conversely, PYR, a slow-acting antimalarial, delayed parasite development beyond 24 h, with no apparent parasite clearance or death within the first 24 h of drug exposure (Figure 3B, PYR). Compound **5** exhibited a morphological development profile comparable to that of the fast-acting inhibitor ART, with detectable pyknotic nuclei observed within the first 24 h. These findings support the fast-acting inhibition by **5.**

### Thio-phosphoranes Act in the Ring and Trophozoite Stages of the Parasite

To further investigate into the parasitological profile of **5**, a specific stage of action assay was conducted. In this assay, highly synchronized parasites at the ring stage were exposed to **5** for specific time frames ranging from 0–8, 8–16, 16–24, 24–32, and 32–40 h posterythrocyte infection.^28^ The aim was to evaluate the antiplasmodial efficacy of the inhibitor at different stages of parasite development, including early ring (0–8 h), late ring (8–16 h), early trophozoite (16–24 h), late trophozoite (24–32 h), and schizont (32–40 h) stages (Figures 4 and S6). Figure 4A illustrates the stage of action profile of **5**, revealing a 10-fold increase in IC_50_ values across all parasite stages. This suggests that exposure to **5** for longer than 8 h was needed for effective inhibition. Consequently, extended exposure time frames of 0–16, 16–32, and 32–40 h were used to assess the inhibitory activities on ring, trophozoite, and schizont forms, respectively (Figure 4B). Given that the IC_50_ values obtained were approximately 5 to 8 times greater than the observed IC_50_ value at 72 h, the results suggest a tendency for **5** to act similarly on ring and trophozoite stages of the parasite. This observation aligns with the requirement for longer exposure times. Overall, these findings corroborate the conclusion that compound **5** operates as a fast-acting inhibitor.

**Figure 4:**
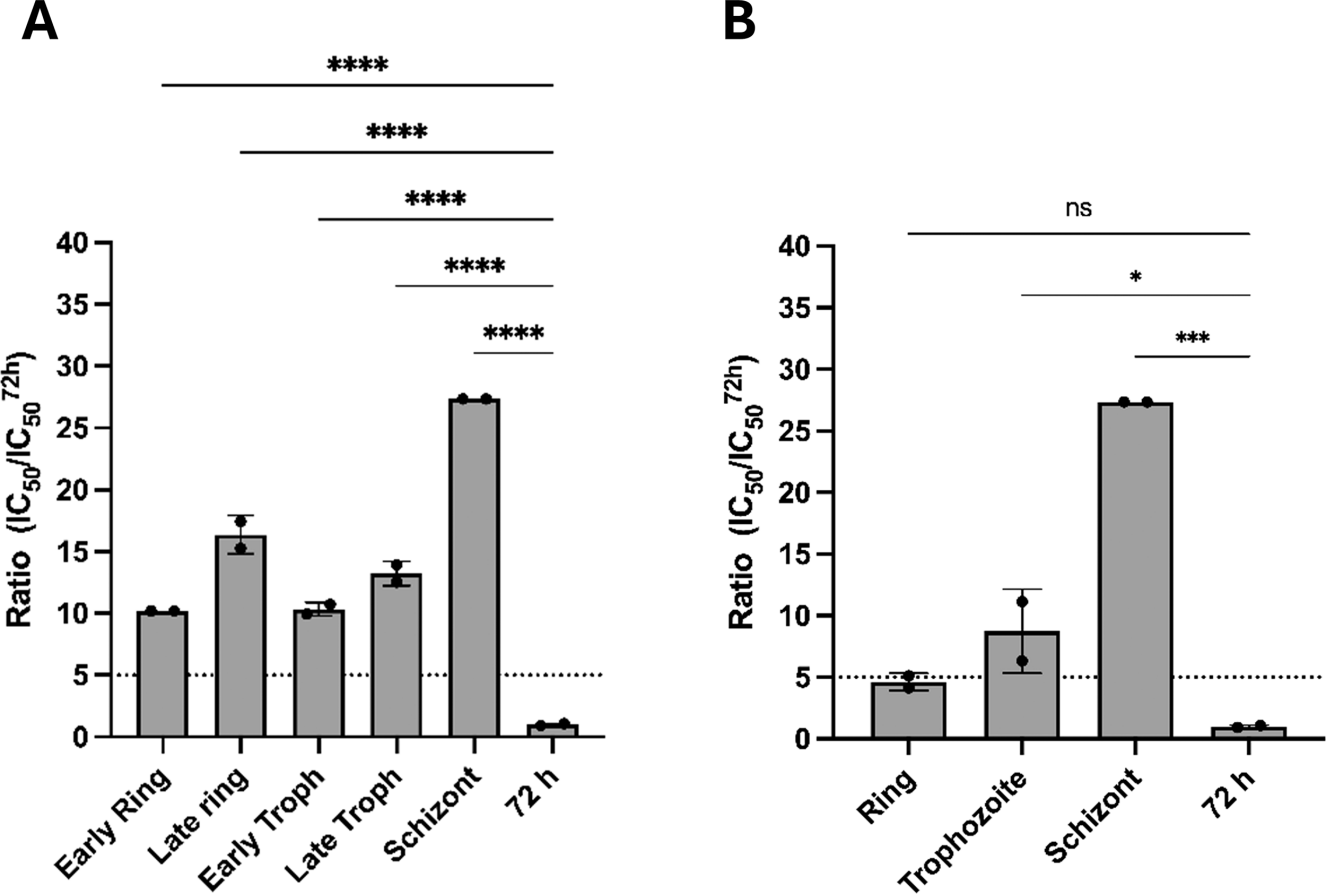
Stage of action assessment of 5. (A) IC_50_ ratios were determined at 8 h of inhibitor pressure at different developmental stages (early ring, late ring, early trophozoite, late trophozoite, and schizont) of *P. falciparum* parasites. (B) IC_50_ ratios were determined at 16 h of inhibitor pressure at different developmental stages (ring, trophozoite) and 8 h for the schizont stage of *P. falciparum* parasites. Data show mean ± standard deviation (N,*n* = 2,2). (ns: not significant; *p < 0.05; ***p < 0.001; ****p < 0.0001).

### Thio-phosphoranes Slightly Increase the Survival Rates of *P. berghei* Infected Mice

To evaluate the efficacy and safety of the chalcogen-phosphorane series in an in vivo malaria model, mice infected with *P. berghei* parasites (NK65 strain) were used. Three infected mice were orally treated with 50 mg/kg of **5** over two consecutive days. Parasitemia was assessed on days 5, 8, and 11 postinfection (Figure 5A). Chloroquine (CQ), used as a positive control, was given at 20 mg/kg for three consecutive days. Treatment with **5** did not result in reduced parasitemia on days 5 and 8, but a modest decline of 13% in parasitemia was noted on day 11. Although the antimalarial effect was limited, an improvement in survival rates was observed in the **5**-treated group compared to the untreated control (Figure 5B). Furthermore, no apparent signs of toxicity, distress or changes in body weight were observed in the animals treated with compound **5** (Figure S7), suggesting that the compound was well tolerated at the tested dose. Collectively, these findings suggest that **5** demonstrates limited efficacy but maintains a favorable safety profile within the murine malaria model.

**Figure 5:**
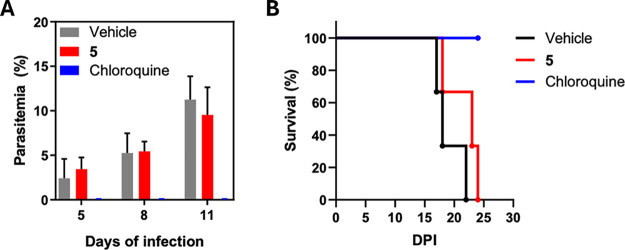
(A) Percentage of parasitemia on days 5, 8, and 11 post infection. Compound 5 was orally administered at a dose of 50 mg/kg, and chloroquine (CQ) was used as a positive control at 20 mg/kg. (B) In vivo survival after treatment with 5, CQ, and vehicle control. Three mice were allotted to each treatment group.

## Conclusions

In this study, we conducted a thorough assessment of the antiplasmodial properties exhibited by the chalcogen-phosphorane series. The findings outlined here serve as a valuable resource for further exploration of this series as potential candidates for hit-to-lead drug discovery. Among the compounds evaluated, compound **5** emerged as the most potent inhibitor, demonstrating low cytotoxic effects on liver and kidney cells. Consequently, compound **5** was chosen as the representative compound for subsequent in vitro and in vivo studies. Compound **5** is a fast-acting inhibitor of *P. falciparum* parasites, particularly targeting the early stages of development. Furthermore, when combined with artesunate, compound **5** demonstrated an additive profile. Importantly, all tested derivatives displayed no cross-resistance against a representative panel of resistant *P. falciparum* strains, suggesting promising potential in overcoming resistance mechanisms. However, the in vivo evaluation revealed that **5** did not effectively reduce parasitemia in a murine malaria model. Nevertheless, a protective effect was observed in terms of survival rates among the group treated with compound **5**. Despite the encouraging antiplasmodial profile exhibited by **5**, medicinal chemistry efforts are necessary to improve both the pharmacodynamic and pharmacokinetic properties of this series. Such efforts are crucial for optimizing the therapeutic potential of chalcogen-phosphoranes as promising candidates for future antimalarial drug development.

## Methods

### Synthesis

The synthesis of compounds **1**–**8** has been previously described.^11^ The HPLC-PAD chromatogram of compounds **1**, **3**, and **5** are indicated in Figure S8.

### In Vitro *P. falciparum* Culture Maintenance

*P. falciparum* chloroquine-sensitive (3D7) and -resistant (Dd2, TM90C6B and 3D7^R^_MMV848) strains, obtained from the BEI source, were maintained using the protocols described elsewhere.^29,30^

### In Vitro SYBR Green I Viability Assay against Asexual *P. falciparum* Stages

The parasite culture was diluted to 0.5% of parasitemia and 2% of hematocrit and 180 μL were added to each well of a 96-well plate previously prepared with 20 μL of the compound at 10-times concentration in a serial dilution. Artesunate was used as an internal control for inhibition, and the plates were kept under a controlled atmosphere (90% N_2_, 5% O_2_ and 5% CO_2_), in a humidified chamber at 37 °C for 72 h. Control wells were prepared in parallel, including negative controls with nonparasitized erythrocytes and positive controls with iRBCs in the absence of compounds. The detailed protocol is described elsewhere.^31,32^

### Hepatocarcinoma Cell and Human Embryo Kidney Cell Culture and Cytotoxicity Evaluation

Hepatocarcinoma cells (HepG2) were cultivated in incomplete RPMI-1640 medium (25 mM HEPES pH 7.4, 21 mM sodium bicarbonate, 22 mM d-glucose, 10 mg/L Hypoxanthine and 25 mg/L Gentamicin) supplemented with 10% (v/v) fetal bovine serum. Human embryonic kidney cells (HEK293) were cultivated in incomplete RPMI-1640 medium (25 mM HEPES pH 7.4, 21 mM sodium bicarbonate, 1 g/L d-glucose, 10 mg/L hypoxanthine and 25 mg/L gentamicin) supplemented with 10% (v/v) fetal bovine serum. Cells were cultivated at 37 °C and 5% CO_2_; the supplemented medium was changed every 2 or 3 days. The detailed protocol is described elsewhere.^32^

### Speed-of-Action

A synchronized *P. falciparum* culture at the ring stage was prepared at 0.5% parasitemia and 2% hematocrit, and an aliquot was dispensed into a 96-well plate. Test compounds were added at a concentration corresponding to 10xIC_50_ for 24 h. Plates were incubated at 37 °C in a controlled atmosphere (90% N_2_, 5% O_2_, 5% CO_2_) and humidified chamber for a total of 72 h. A negative control consisting of untreated infected erythrocytes was included to allow normal parasite development, and additional control wells were maintained with the antimalarials artesunate and pyrimethamine. Blood smears were collected at 0, 24, 48, and 72 h, Giemsa-stained, and examined to assess parasite survival and morphological progression relative to the controls.

For the quantitative assay, a ring-stage *P. falciparum* culture was prepared at 0.5% parasitemia and 2% hematocrit and distributed into three 96-well plates. Each plate was exposed to the compounds for different time-points (24, 48, or 72 h). After treatment, the plates corresponding to the 24 and 48 h time points were washed twice with fresh medium and incubated for an additional 48 or 24 h, respectively. At the end of the 72 h period, all plates were analyzed using the SYBR Green I assay to determine compound inhibitory activity at each time point.

### Stage of Action

To determine the specific stage of action of **5**, parasite growth was evaluated after incubation with serial dilutions of **5** for specific time intervals of 0–8, 8–16, 16–24, 24–32, and 32–40 h after invasion. Six 96-well plates were prepared with tightly synchronized parasites, using magnetic columns, in the ring stage right after erythrocyte invasion. After the exposure time, iRBC were washed twice with RPMI-1640 medium and a serial dilution of drugs was added to the following plate time interval. Five plates were used to evaluate the antiplasmodial potency of **5** and one plate was prepared for a 72 h assay. At 60 h after invasion for the five plates, the supernatant was removed and 100 μL of PBS was added, resuspended and 100 μL of Lysis Buffer containing 0.002% (v/v) SYBR Green I was added and incubated for 30 min followed by reading in a SpectraMAX Gemini EM plate reader (Molecular Devices Corp., Sunnyvale, CA) with excitation at 485 nm, and emission at 535 nm. The half maximal inhibitory concentration (IC_50_) was determined by nonlinear regression analysis of the resulting concentration–response curve using the GraphPad Prism 8 software. The detailed protocol is described elsewhere.^32^

### Antimalarial Drug Combination Assay

Compound **5** was combined with artesunate in molar ratios of 7:0, 6:1, 5:2, 4:3, 3:4, 2:5, 1:6, and 0:7 (artesunate:**5**) for the construction of the isobolograms, based on the individual IC_50_ values that were previously determined by the SYBR Green I assay. Additivity ranges were considered in the isobologram analysis, following Grabovsky and Tallarida’s method.^33^ The detailed protocol is described elsewhere.^32^

### Cross-Resistance Assessment

The antiplasmodial activity of **1**, **3**, and **5** was assessed against a panel of *P. falciparum* strains: 3D7 (chloroquine-sensitive), Dd2 (resistant to chloroquine, mefloquine, sulfadoxine, and pyrimethamine), TM90C6B (resistant to chloroquine, sulfadoxine, pyrimethamine, and atovaquone), and 3D7^R^_MMV848 (resistant to MMV692848, a PI4K inhibitor). The assay to determine the IC_50_ value of **1**, **3**, and **5** against the panel of resistant strains was carried out as described above. After the determination of the IC_50_ value for each resistant strain, using the procedure described earlier, a resistance index (RI) was calculated as the ratio of the IC_50_ value in the resistant to that in the sensitive strain.

### In Vivo Assay

The suppressive test was performed as described by Peters with some modifications.^34^ The *P. berghei* NK65 strain was obtained as a donation from the BEI source and maintained through weekly blood passages. For the experiments, groups of up to three mice were inoculated *ip* with 1 × 10^6^ infected erythrocytes, kept together for about 4 h, and then randomly distributed into groups up to four mice per cage. The mice were treated daily for two consecutive days with compounds freshly diluted in bovine serum and administered orally at 50 mg/kg; the control groups received either the drug vehicle or the antimalarial CQ administered at 20 mg/kg for three consecutive days, diluted in distilled water. On days 5, 8, and 11 after the parasite inoculation, blood was taken from the tail of each mouse and used to prepare thin smears that were methanol-fixed, Giemsa-stained, and examined microscopically (1000×) to determine parasitemia. The use of laboratory animals was approved by the Ethic Committee on Animal Use of the Federal University of Sao Paulo (CEUA/UNIFESP 3004230222). During the investigation, animals were monitored each day beginning on day 11, and evaluated based on established compassionate end point standards. These standards included changes in body mass and consumption of food or water; noticeable physical signs (such as closed eyes, eye or nose secretions, unkempt or tousled coats, and a hunched stance); medical symptoms (like difficulty breathing, shifts in heart rate and breathing patterns, changes in bowel movements and body heat); spontaneous behavior alterations (including vocal outbursts or self-harm); as well as behavioral reactions (for instance, heightened aggression).^35^ All facility and national protocols regarding the handling and use of laboratory animals were adhered to. The detailed protocol is described elsewhere.^32^

## Supplementary Material



## Data Availability

The data underlying this study are available in the published article and its online Supporting Information.

## Notes

Concentration–response curve of all eight compounds evaluated against the *P. falciparum* 3D7 strain, human hepatocellular carcinoma cells (HepG2), human embryo kidney cells (HEK293), standard antimalarials (artesunate, pyrimethamine, atovaquone, and MMV692848), and compounds **1**, **3**, and **5** against sensitive (3D7 strain) and resistant (Dd2, TM90C6B, and 3D7^R^_MMV848 strains) strains; HPLC-PAD chromatogram of compounds **1**, **3**, and **5**; and hemolytic activity of compounds **1**, **3**, and **5** (PDF)

## Abbreviations

ACT: artemisinin combination therapy
ART: artesunate, ATV, atovaquone
CC50: cytotoxic concentration of an inhibitor that is required for 50% inhibition in vitro
CQ: chloroquine
∑FIC50: sum of the fraction concentration of a drug that is required for 50% inhibition in vitro
HEPES: 2-[4-(2-hydroxyethyl)­piperazin-1-yl]­ethanesulfonic acid
HepG2: hepatocellular carcinoma cells
HEK293: human embryo kidney 293 cells
IC50: concentration of a drug that is required for 50% inhibition in vitro
*ip*: intraperitoneal
iRBCs: infected red blood cells
MTT: 3-(4,5-dimethyl thiazol-2-yl)-2,5-diphenyltetrazolium bromide
ND: not determined
P. berghei: *Plasmodium berghei*
Pb: *Plasmodium berghei*
P. falciparum: *Plasmodium falciparum*
Pf: *Plasmodium falciparum*
PYR: pyrimethamine
RBC: red blood cell
RI: resistance index
RPMI 1640: Roswell park memorial institute medium
SD: standard deviation
SI: selectivity index.
