# Supplementary material for: Fast-Acting Chalcogen-Phosphoranes Inhibit Sensitive and Resistant Plasmodium falciparum Strains
Source: ACS Omega. 2026 Jan 5;11(2):2842–50. doi: 10.1021/acsomega.5c08794 (PMC12824749; doi:10.1021/acsomega.5c08794)
Supplement: Supplementary file 1 [file ao5c08794_si_001.pdf]

Supporting Information

For

# Fast-Acting Chalcogen-Phosphoranes Inhibit Sensitive and Resistant Plasmodium falciparum Strains

*Igor M. R. Moura,[a] Camila S. Barbosa,[b] Giovana Rossi Mendes,[a] Anna Caroline Campos Aguiar,[b] Fabio C. Cruz,[c] Paulo Henrique Menezes,[d] Rafael Victorio Carvalho Guido*[a]*

[a] São Carlos of Physics Institute, University of São Paulo (USP), 13566-590, São Carlos, SP, Brazil.

[b] Department of Microbiology, Immunology and Parasitology, Federal University of São Paulo (UNIFESP), Escola Paulista de Medicina, 04023-062, São Paulo, SP, Brazil

[c] Department of Pharmacology, Federal University of São Paulo (UNIFESP), Escola Paulista de Medicina, 04023-062, São Paulo, SP, Brazil.

[d] Department of Fundamental Chemistry, Federal University of Pernambuco, 50740-560, Recife, PE, Brazil.

* Corresponding authors: pauloh.menezes@ufpe.br ; rvcguido@usp.br

**List of Supplementary Figures**

**Figure S1:** Representative concentration-response curve of all eight compounds evaluated against P. falciparum 3D7 strain. **A**) compound **1**, **B**) compound **2**, **C**) compound **3**, **D**) compound **4**, **E**) compound **5**, **F**) compound **6**, **G**) compound **7**, and **H**) compound **8**. Values show mean ± SD. N,n = 2,2 ........ S4

**Figure S2:** Representative concentration-response curve of all six compounds evaluated against human hepatocellular carcinoma cells (HepG2). **A**) compound **1**, **B**) compound **2**, **C**) compound **3**, **D**) compound **4**, **E**) compound **5**, **F**) compound **8**. Values show mean ± SD. N,n = 2,2.......... S5

**Figure S3:** Representative concentration-response curve of all six compounds evaluated against human embryo kidney cells (HEK293). **A**) compound **1**, **B**) compound **2**, **C**) compound **3**, **D**) compound **4**, **E**) compound **5**, **F**) compound **8**. Values show mean ± SD. N,n = 2,2 .................. S6

**Figure S4:** Hemolytic activity of compounds **1**, **3** and **5** tested at 20 µM and 2 µM in fresh human red blood cells after 72 h of incubation. Saponin (0.1%) and DMSO (0.02%) were used as positive and vehicle controls, respectively. Values show mean ± SD. N,n = 2,4 ..................................... S7

**Figure S5:** Representative concentration-response curves of compounds **1** (**A**), **3** (**B**), **5** (**C**) and the standard antimalarials artesunate (**D**), atovaquone (**E**), pyrimethamine (**F**), and MMV692848 (**G**) against the sensitive (3D7 = black) and resistant (Dd2 = yellow, TM90C6B = blue, and 3D7R_MMV848 = pink) strains tested. Values show mean ± SD. N,n = 2-3,2 .......................... S7

**Figure S6:** Stage of action assessment of **5**. (**A**) $IC_{50}$ ratios were determined at 8 h of inhibitor pressure at different developmental stages (early ring, late ring, early trophozoite, late trophozoite, and schizont) of *P. falciparum* parasites. (**B**) $IC_{50}$ ratios were determined at 16 h of inhibitor pressure at different developmental stages (ring, trophozoite) and 8 h for the schizont stage of *P. falciparum* parasites. Data show mean ± standard deviation (N,n = 2,2). (ns: not significant; *p < 0.05; **p < 0.005; ***p < 0.001; ****p < 0.0001). .......................................................... S8

**Figure S7:** HPLC-PAD chromatogram of compounds **1** (**A**), **3** (**B**), and **5** (**C**). ....................... S10

**Figure S8:** Average body weight (mean ± SD) of mice treated with vehicle (black line), chloroquine (yellow line), and compound **5** (blue line) up to day 11. ......................................... S9

## 1. Experimental Procedures

### 1.1 HPLC analysis

HPLC analyses were carried out on a Waters Alliance 2695 system equipped with a Waters 2996 photodiode array detector, and a Micromass ZQ2000 single quadrupole mass spectrometer (MS) with an electrospray ionization (ESI) interface.

Chromatographic separations were performed on a Waters X-Terra RP 18 column (4.6 × 250 mm, 5 µm particle size) using a mobile phase flow rate of 1 $mL \cdot min^{-1}$. The mobile phase consisted of (A) acetonitrile with 0.1% formic acid, (B) methanol with 0.1% formic acid and (C) water with 0.1% formic acid. The gradient program was as follows: 0–1.0 min, 5% A, 5% B and 90% C; 1.0–21.0 min, linear gradient to 50% A and 50% B; 21.0–23.0 min, hold at 50% A and 50% B; and 23.0–30.0 min, re-equilibration at 5% A, 5% B and 90% C. The total run time was 30 min, with an injection volume of 15 µL. System pressure limits were set between 0 and 5000 psi, with a maximum operating pressure of 2200 psi during elution.

The PDA detector acquired UV spectra at 254 nm. The mass spectrometer was operated in ESI positive mode under the following conditions: capillary voltage, 3.0 kV; source block temperature, 100 °C; desolvation temperature, 350 °C; cone gas flow, 50 $L \cdot h^{-1}$; and desolvation gas flow, 350 $L \cdot h^{-1}$, both supplied by a Nitrogen Peak Scientific N110DR source. The MS detection range was set to m/z 120–1100 with total ion current acquisition.

Data acquisition and processing were conducted using Empower 2.0 software.

### 1.2 Hemolytic Assay

Fresh human red blood cells (RBCs) were employed to evaluate hemolytic activity. RBCs were incubated with compounds **1**, **3** and **5** (20 µM and 2 µM), saponin (0.1%), or DMSO (0.02%) in 96-well plates at 37°C, using a 2% hematocrit suspension. Hemolysis was assessed at 72 h of incubation. As controls, RBCs incubated with RPMI medium alone served as the negative control, while 0.1% saponin was used as the positive control. Following the incubation period, the plates were centrifuged, and the supernatant was transferred to new plates for hemoglobin quantification by measuring absorbance at 540 nm.

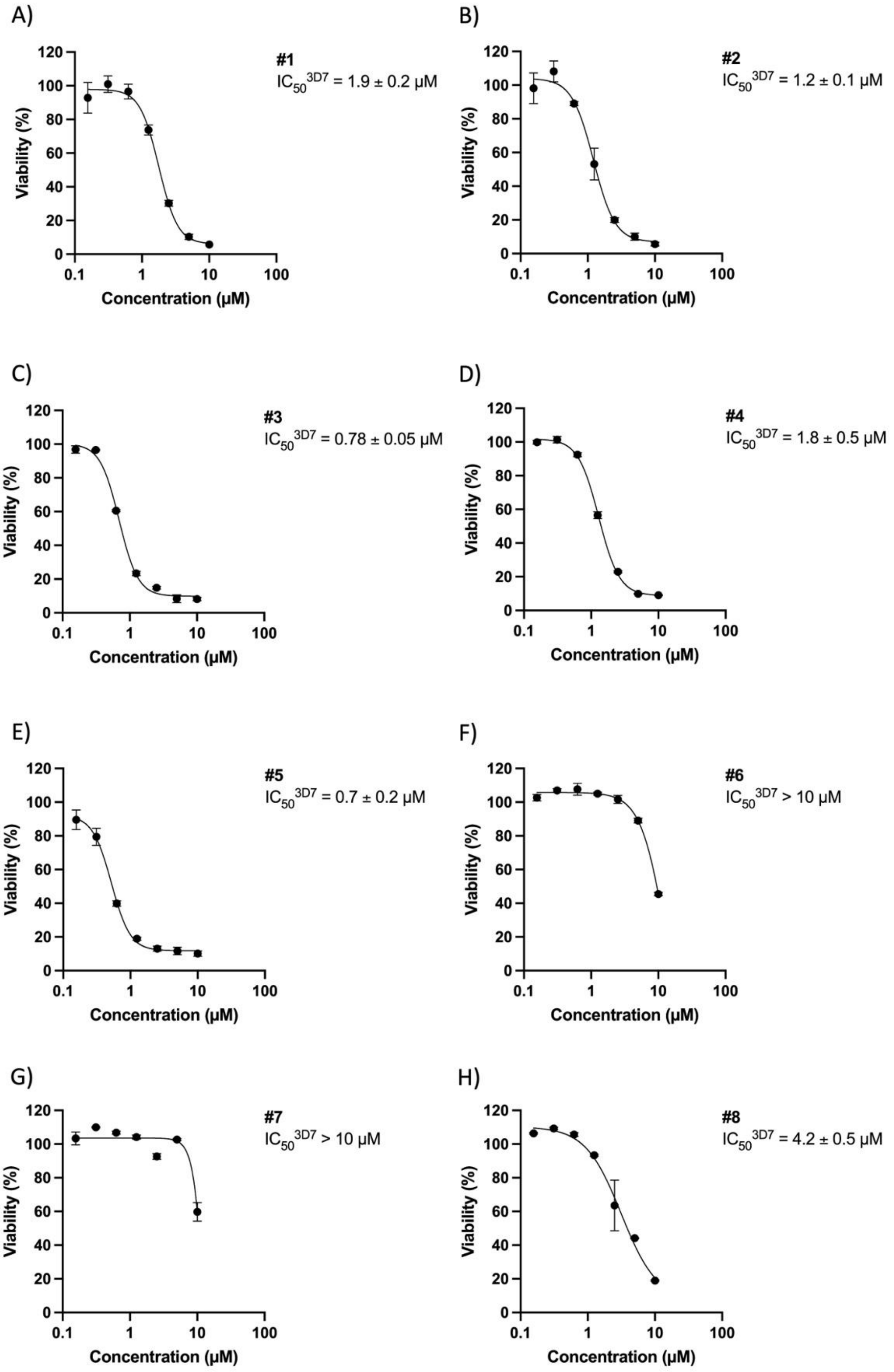


**Figure S1:** Representative concentration-response curve of all eight compounds evaluated against *P. falciparum* 3D7 strain. **A**) compound **1**, **B**) compound **2**, **C**) compound **3**, **D**) compound **4**, **E**) compound **5**, **F**) compound **6**, **G**) compound **7**, and **H**) compound **8**. Values show mean ± SD. N,n = 2,2

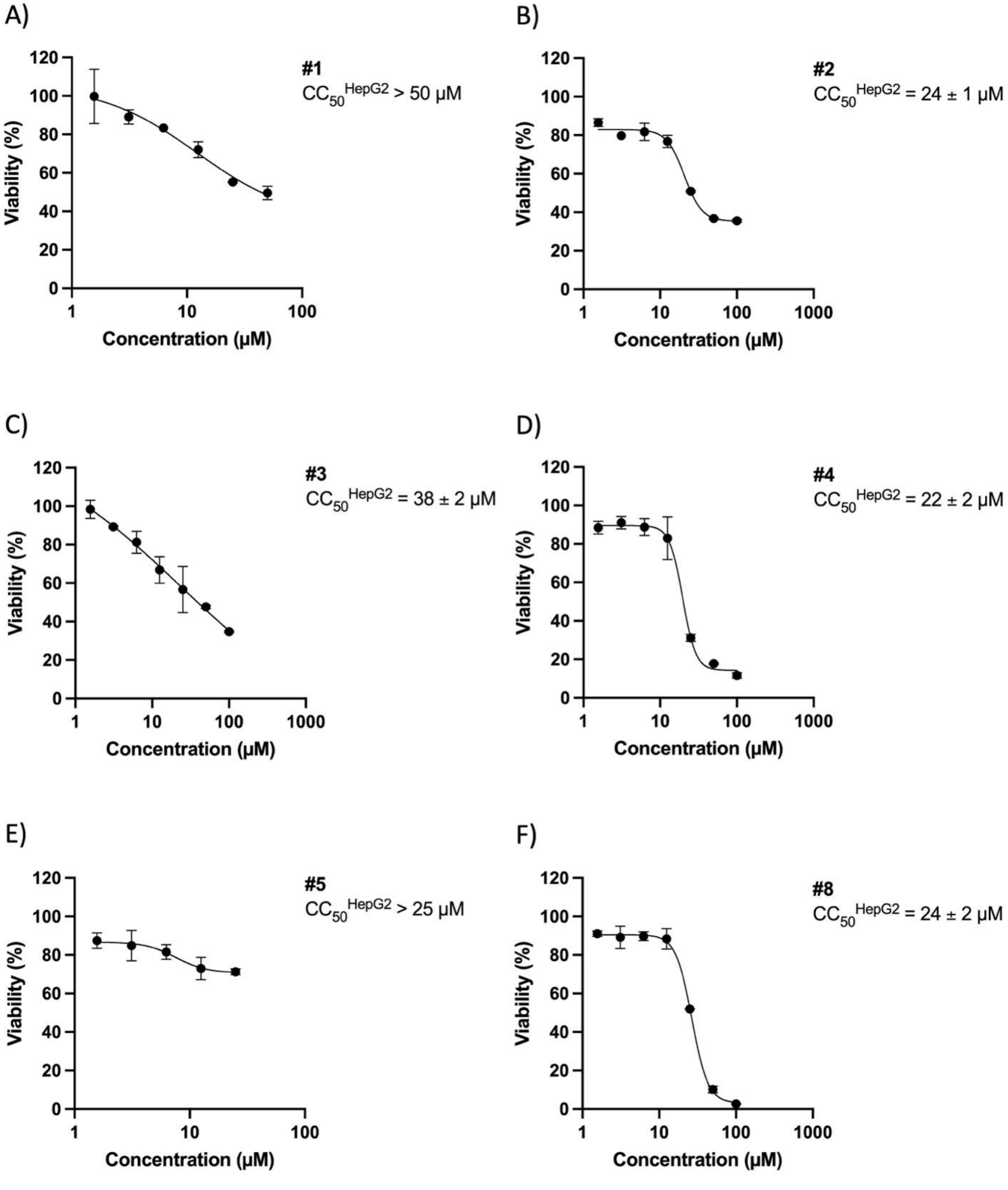


**Figure S2:** Representative concentration-response curve of all six compounds evaluated against human hepatocellular carcinoma cells (HepG2). **A**) compound **1**, **B**) compound **2**, **C**) compound **3**, **D**) compound **4**, **E**) compound **5**, **F**) compound **8**. Values show mean ± SD. N,n = 2,2

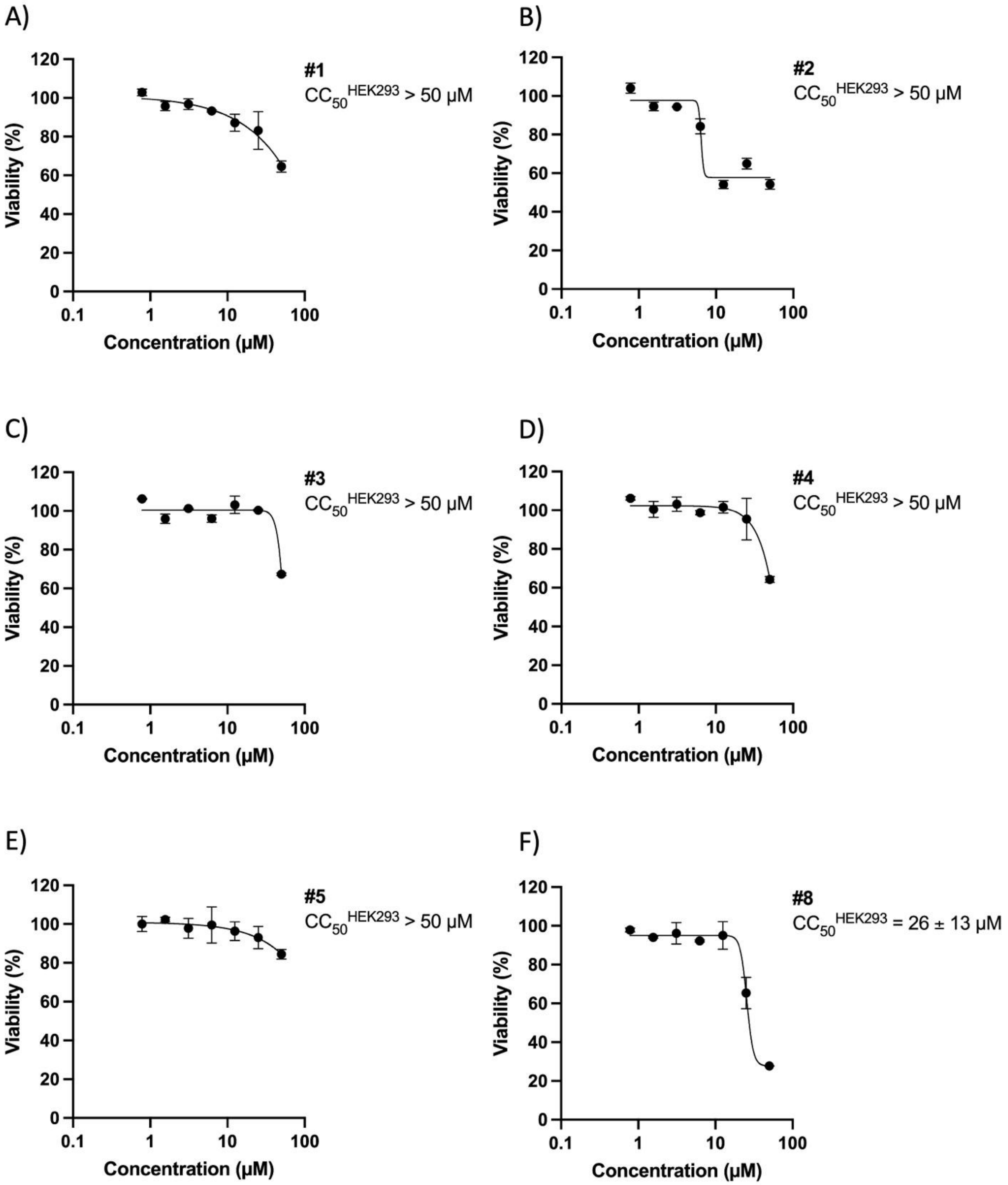


**Figure S3:** Representative concentration-response curve of all six compounds evaluated against human embryo kidney cells (HEK293). **A**) compound **1**, **B**) compound **2**, **C**) compound **3**, **D**) compound **4**, **E**) compound **5**, **F**) compound **8**. Values show mean ± SD. N,n = 2,2

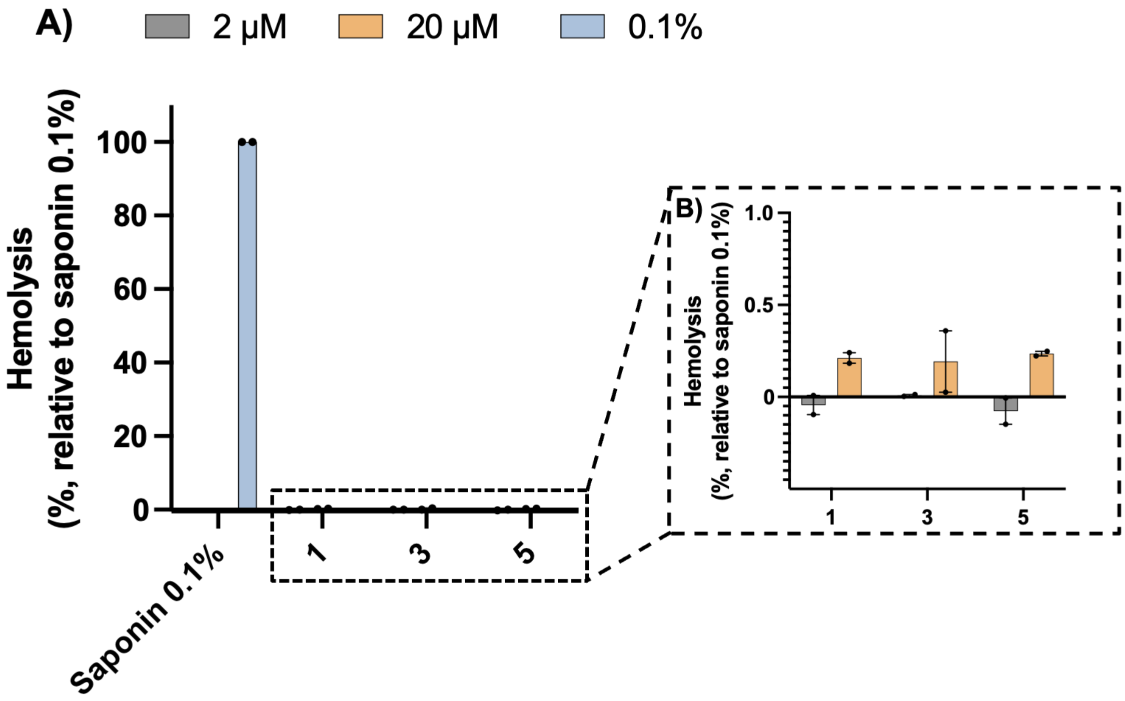


**Figure S4:** Hemolytic activity of compounds **1**, **3** and **5** tested at 20 µM and 2 µM in fresh human red blood cells after 72 h of incubation. Saponin (0.1%) and DMSO (0.02%) were used as positive and vehicle controls, respectively. Values show mean ± SD. N,n = 2,4

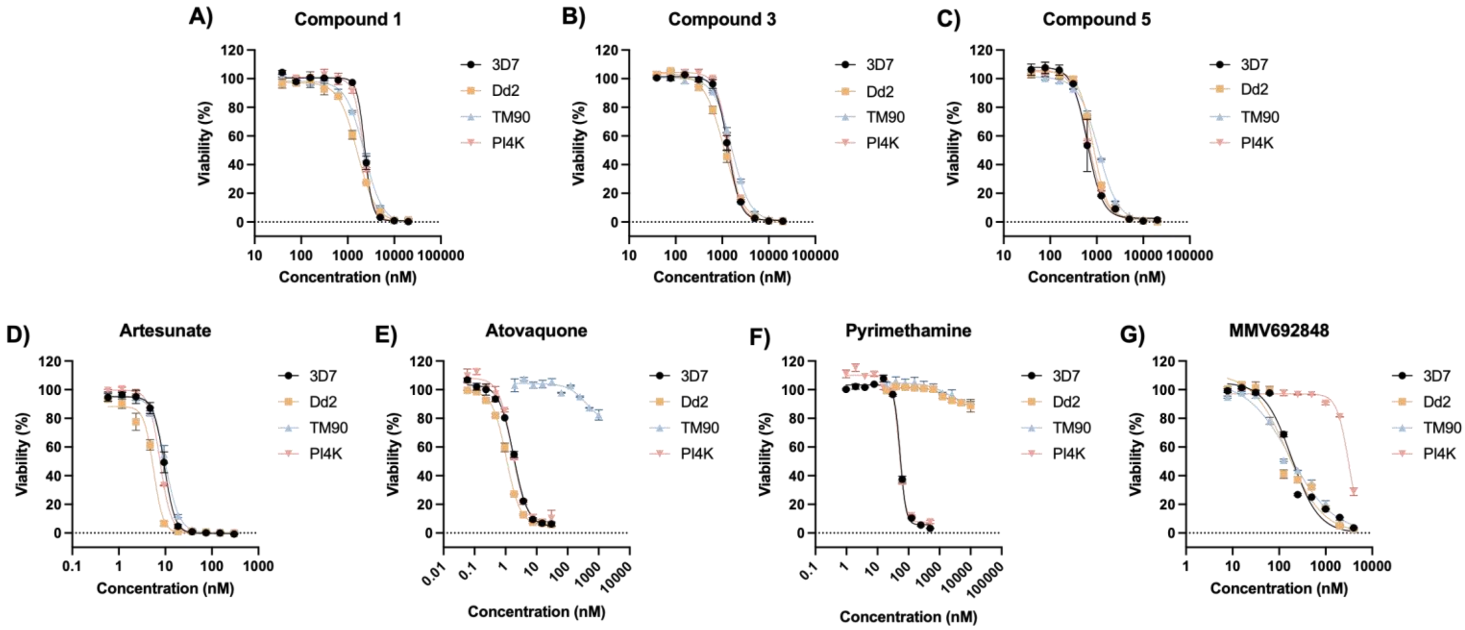


**Figure S5:** Representative concentration-response curves of compounds **1** (**A**), **3** (**B**), **5** (**C**) and the standard antimalarials artesunate (**D**), atovaquone (**E**), pyrimethamine (**F**), and MMV692848 (**G**) against the sensitive (3D7 = black) and resistant (Dd2 = yellow, TM90C6B = blue, and 3D7R_MMV848 = pink) strains tested. Values show mean ± SD. N,n = 2-3,2

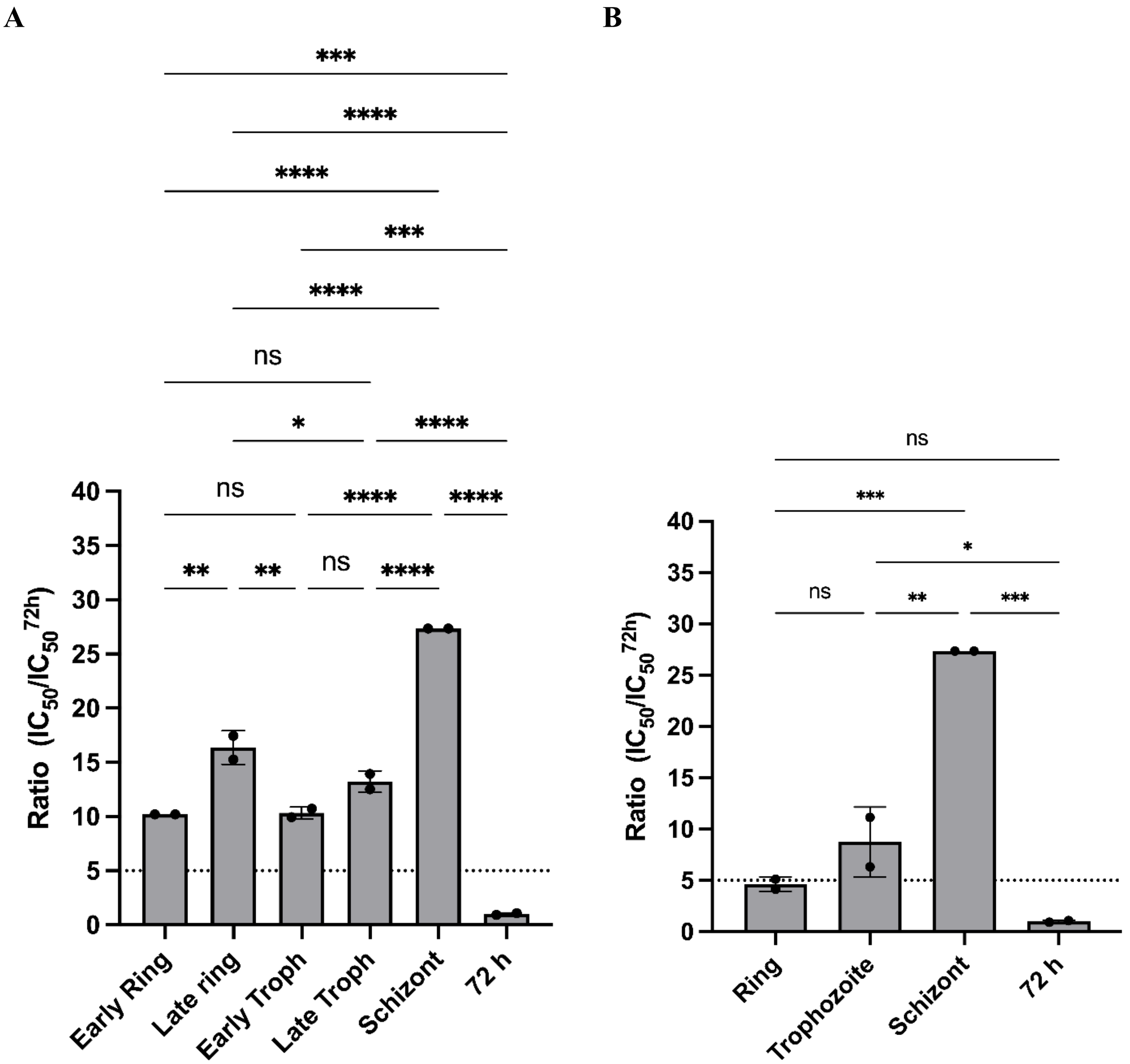


**Figure S6:** Stage of action assessment of **5**. (**A**) $IC_{50}$ ratios were determined at 8 h of inhibitor pressure at different developmental stages (early ring, late ring, early trophozoite, late trophozoite, and schizont) of *P. falciparum* parasites. (**B**) $IC_{50}$ ratios were determined at 16 h of inhibitor pressure at different developmental stages (ring, trophozoite) and 8 h for the schizont stage of *P. falciparum* parasites. Data show mean ± standard deviation (N,n = 2,2). (ns: not significant; *$p < 0.05$; **$p < 0.005$; ***$p < 0.001$; ****$p < 0.0001$).

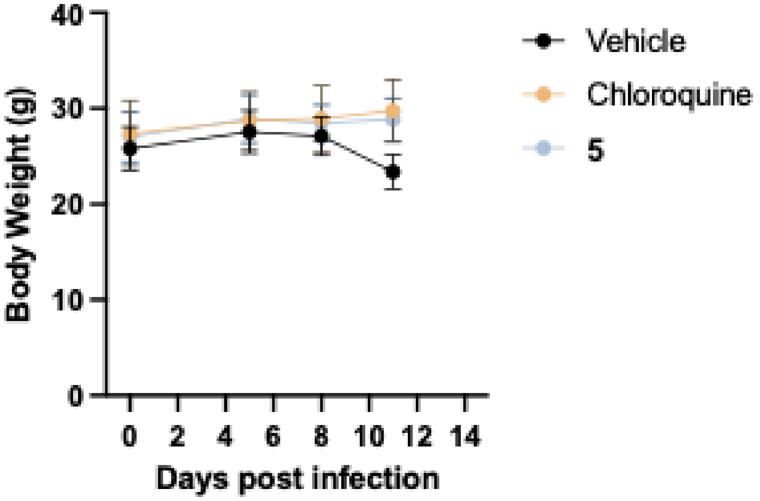


**Figure S7:** Average body weight (mean ± SD) of mice treated with vehicle (black line), chloroquine (yellow line), and compound **5** (blue line) up to day 11.

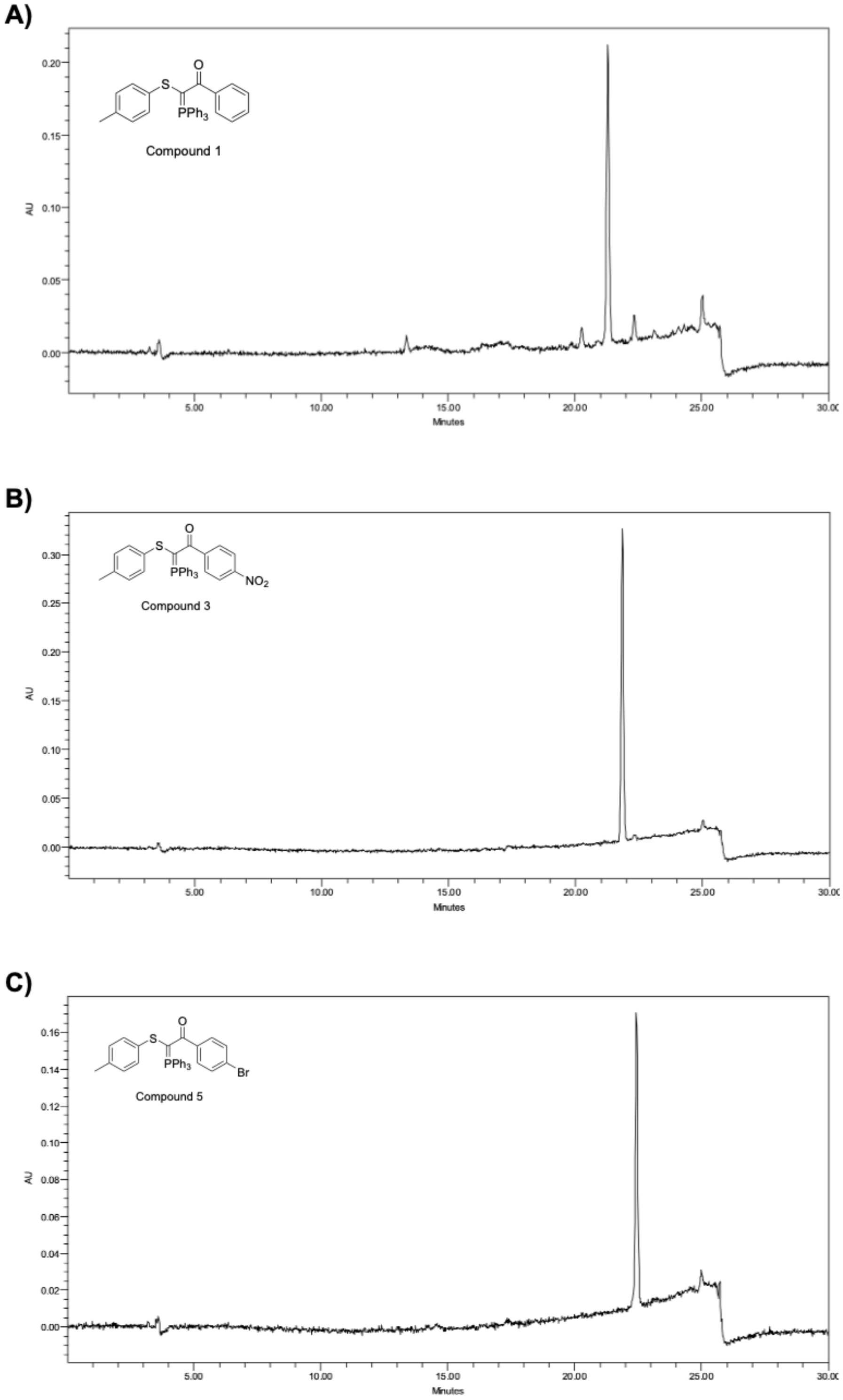


**Figure S8:** HPLC-PAD chromatogram of compounds **1** (**A**), **3** (**B**), and **5** (**C**).
